# Maternal 12-HETE is associated with childhood asthma and the responses to prenatal omega-3 supplementation

**DOI:** 10.1016/j.xcrm.2026.102689

**Published:** 2026-03-17

**Authors:** Liang Chen, Nicklas Brustad, Jonathan Thorsen, Tingting Wang, Mina Ali, Julie N. Kyvsgaard, Mario Lovric, Parvaneh Ebrahimi, Yang Luo, Casper-Emil T. Pedersen, Nicole Prince, Rachel S. Kelly, Ann-Marie M. Schoos, Nilo Vahman, Morten A. Rasmussen, Susanne Brix, Augusto A. Litonjua, Scott T. Weiss, Craig E. Wheelock, Jessica Lasky-Su, Klaus Bønnelykke, Jakob Stokholm, Bo Chawes

**Affiliations:** 1 COPSAC, Copenhagen Prospective Studies on Asthma in Childhood, Copenhagen University Hospital - Herlev and Gentofte, Copenhagen, Denmark; 2 Department of Clinical Medicine, Faculty of Health and Medical Sciences, University of Copenhagen, Copenhagen, Denmark; 3 Department of Pediatrics, Slagelse Hospital, Slagelse, Denmark; 4 Food Microbiology, Gut Health, and Fermentation, Department of Food Science, University of Copenhagen, Copenhagen, Denmark; 5 Centre for Applied Bioanthropology, Institute for Anthropological Research, Zagreb, Croatia; 6 Faculty of Electrical Engineering, Computer Science and Information Technology Osijek, Josip Juraj Strossmayer University of Osijek, Osijek, Croatia; 7 Channing Division of Network Medicine, Department of Medicine, Brigham and Women’s Hospital and Harvard Medical School, Boston, MA, USA; 8 Department of Biotechnology and Biomedicine, Technical University of Denmark, Kgs. Lyngby, Denmark; 9 Division of Pediatric Pulmonary Medicine, Golisano Children’s Hospital, University of Rochester Medical Center, Rochester, NY, USA; 10 Unit of Integrative Metabolomics, Institute of Environmental Medicine, Karolinska Institute, Stockholm, Sweden; 11 Department of Respiratory Medicine and Allergy, Karolinska University Hospital, Stockholm, Sweden

**Keywords:** 12-hydroxyeicosatetraenoic acid, 12-HETE, oxylipins, prenatal omega-3 supplementation, n-3 LCPUFA, childhood asthma, respiratory infections, infant airway microbiome, airway immune profiles, precision prevention, COPSAC, VDAART

## Abstract

A recent mouse study has shown that deficiency in 12-hydroxyeicosatetraenoic acid (12-HETE) affects neonatal alveolar macrophage imprinting and associates with increased respiratory morbidity, but this has not been investigated in humans. Utilizing data from two mother-child cohorts, COPSAC_2010_ and VDAART, we demonstrate that undetectable maternal plasma 12-HETE during pregnancy associates with increased risk of childhood asthma and respiratory infections alongside an altered infant airway microbiota structure and airway immune profile. Further, we observed an interaction between maternal 12-HETE levels and maternal N-3 long-chain polyunsaturated fatty acid (n-3 LCPUFA) supplementation in a randomized clinical trial in COPSAC_2010_ and maternal dietary *n*-3 LCPUFA intake in VDAART in relation to offspring respiratory morbidity; higher prenatal n-3 LCPUFA exposure reduced asthma and respiratory infection among mothers with detectable 12-HETE levels. These findings identify maternal 12-HETE as a potential biomarker for risk of offspring respiratory morbidity and suggest that maternal 12-HETE status may determine responsiveness to prenatal n-3 LCPUFA supplementation.

## Highlights

• Maternal 12-HETE levels associate with childhood asthma risk
• Maternal 12-HETE levels relate to infant airway microbiota and immune patterns
• Higher prenatal n-3 LCPUFA intake reduced childhood asthma risk in offspring
• Effect of prenatal n-3 LCPUFA intake varies by maternal 12-HETE status

## Graphical abstract

**Figure:**
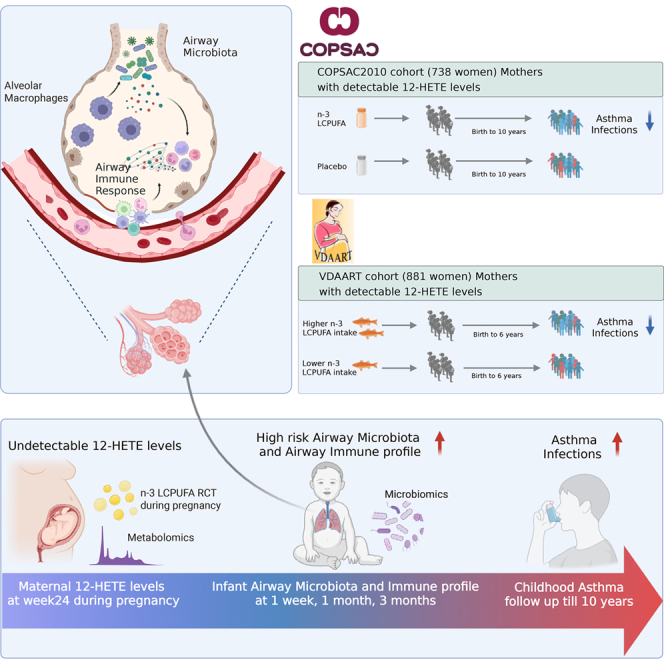

## Teaser

Across two birth cohorts, Liang et al. found that maternal 12-HETE levels during pregnancy associate with childhood asthma and respiratory infections alongside altered infant airway microbiota and immune patterns. Effect of prenatal n-3 LCPUFA intake varies by maternal 12-HETE status, suggesting 12-HETE as a potential biomarker for precision prevention.

## Introduction

Asthma is a multifaceted chronic respiratory disease characterized by airway inflammation, hyperresponsiveness, and variable airflow obstruction, affecting individuals of all ages^1^^,^^2^^,^^3^ and with no known cure, making the prevention of asthma during early life and childhood a potential opportunity to reduce its long-term impact.^4^^,^^5^^,^^6^ Early diagnosis, intervention, and risk factor management during these formative years can significantly improve respiratory health and quality of life.^7^^,^^8^^,^^9^

Emerging evidence suggests that fetal lung growth and neonatal imprinting of alveolar macrophages (AMs) are related to early-life respiratory infections and development of childhood asthma.^10^^,^^11^^,^^12^^,^^13^ AMs are crucial for pulmonary homeostasis and immune surveillance.^14^^,^^15^^,^^16^ Dysregulation of AM function has been implicated in asthma pathogenesis, contributing to aberrant immune responses and airway inflammation.^12^^,^^17^ Unlike interstitial macrophages derived from the bone marrow, AMs originate from fetal liver monocytes, playing essential roles in lung development and maturation during the first week of life.^10^^,^^15^^,^^18^^,^^19^ Eicosanoid lipid mediators are well recognized in the pathobiology of asthma and allergy, and play a significant role in modulating macrophage function^20^^,^^21^ and immune regulation,^22^^,^^23^ particularly in the contexts of asthma and allergy.^24^^,^^25^^,^^26^ Especially, neutrophil-derived 12-hydroxyeicosatetraenoic acid (12-HETE), which is an eicosanoid lipid mediator formed from arachidonic acid (AA) by lipoxygenase (LOX) enzyme activity,^27^^,^^28^ has been shown in a recent mouse study to be important for neonatal imprinting of AMs.^10^ Further, the lack of 12-HETE was associated with compromised AM function and increased susceptibility to respiratory infections, prompting investigation into the relevance of 12-HETE in human respiratory infections and asthma development, which has not been studied so far. AMs serve as the primary defense line in the distal airways, potentially also influencing the resident airway microbiota.^10^^,^^13^^,^^29^ Neonatal bacterial airway colonization and immune perturbations have emerged as key determinants in the origins of childhood asthma, suggesting intricate microbiota-immune interactions in early infancy that predispose to asthma development.^30^^,^^31^^,^^32^^,^^33^ Macrophages carry out a range of essential immune functions locally in the lungs, which can be dysregulated in asthma.^34^ Further, children with poorly controlled asthma have been shown to have compromised AM function characterized by decreased phagocytosis and increased apoptosis.^17^ 12-HETE can also activate BLT2 (leukotriene B4 receptor 2) as a low-affinity agonist, whereas 12-HHT is a high-affinity endogenous ligand.^35^ BLT2 is expressed on airway epithelium and innate immune cells, where it can influence both inflammatory signaling and epithelial barrier repair. In asthma, BLT2 activity appears context dependent, with studies implicating it in airway inflammation, while also supporting barrier-protective effects in epithelial injury settings.^36^^,^^37^^,^^38^

N-3 long-chain polyunsaturated fatty acids (n-3 LCPUFAs) found in fatty fish and fish oil, notably eicosapentaenoic acid (EPA) and docosahexaenoic acid (DHA), have been reported to exhibit anti-inflammatory properties and influence immune cell functions.^39^^,^^40^ In particular, they have been suggested to promote macrophage polarization into an M2 phenotype,^41^^,^^42^ which is associated with resolving inflammation and promoting anti-inflammatory responses.^43^^,^^44^ The G protein-coupled receptor GPR120 has been identified as a receptor for n-3 fatty acids on macrophages and adipose cells, suggesting a potential mechanism for the anti-inflammatory effects of n-3 LCPUFA. In a randomized controlled trial (RCT), we have demonstrated that fish oil-derived n-3 LCPUFA supplementation during pregnancy reduced the risk of recurrent wheeze, asthma, and respiratory infections in childhood.^45^^,^^46^ Further, in the ALSPAC (Avon Longitudinal Study of Parents and Children) and BAMSE (Swedish abbreviation for Children, Allergy, Milieu, Stockholm, Epidemiology) birth cohort studies, higher intake of EPA and DHA from fatty fish in childhood has been associated with a lower risk of asthma up to mid-adolescence among children with specific fatty acid desaturase (FADS) genotypes.^47^ Therefore, we hypothesized that maternal 12-HETE levels are associated with risk of childhood respiratory morbidity through alterations of the airway microbiome and immune development in early childhood and that this can be modified by prenatal n-3 LCPUFA supplementation dependent on maternal 12-HETE levels at the start of supplementation.

In this study, we aimed to investigate the association between maternal 12-HETE levels in plasma during pregnancy and the risk of childhood asthma and respiratory infections in childhood, utilizing infant airway microbiota colonization patterns and airway immune profiles to explore such associations. Additionally, we investigated the potential role of maternal 12-HETE levels in modifying the effect of prenatal n-3 LCPUFA supplementation for reducing the risk of childhood asthma and respiratory infections in the offspring. Unraveling the interplay between prenatal n-3 LCPUFA supplementation, maternal 12-HETE levels, infant airway microbiota and airway immune profiles in relation to respiratory morbidity may offer valuable insights into therapeutic strategies and personalized approaches for primary prevention.

## Results

### Baseline characteristics

Data on 12-HETE levels in plasma samples collected during pregnancy were available for 727 mothers in COPSAC_2010_ at pregnancy week 24 and for 779 mothers in VDAART at pregnancy week 32. A comparison of baseline characteristics among mothers with detectable vs. undetectable 12-HETE levels in pregnancy showed no significant differences in either COPSAC_2010_ or VDAART (Tables S1 and S2).

This was despite the observation that VDAART is a more racially and ethnically diverse population, including more mothers with asthma and higher BMI, lower household income, and lower education levels compared to COPSAC_2010_ (Table S3).

Previous studies have shown that the FADS genotype can modify the effect of n-3 LCPUFA supplementation for prevention of childhood asthma, but we found no significant difference in FADS genotype among mothers with detectable vs. undetectable 12-HETE levels.

### Maternal pregnancy 12-HETE levels vs. childhood asthma and respiratory infections in offspring

**Figure 1:**
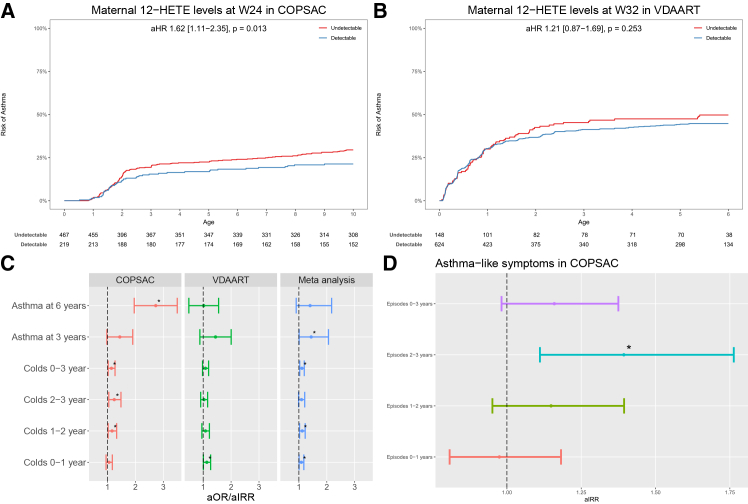
Maternal 12-HETE and offspring respiratory outcomes (A) Kaplan-Meier curves show risk of asthma in children from birth to age 10 years according to maternal detectable vs. undetectable 12-HETE levels during pregnancy in COPSAC_2010_. (B) Kaplan-Meier curves show risk of asthma in children from birth to age 6 years according to maternal detectable vs. undetectable 12-HETE levels during pregnancy in VDAART. (C) Association between maternal 12-HETE levels and early-life asthma/wheeze at age 3 and 6 years, colds from birth to age 3 years in COPSAC_2010_ and VDAART, and meta-analysis across the cohorts. For asthma, Logistics regression, ∗*p* < 0.05; for colds, quasi-Poisson regression analysis, ∗*p* < 0.05. (D) Association between maternal 12-HETE levels and episodes with asthma-like symptoms from birth to age 3 years in COPSAC_2010_. Quasi-Poisson regression analysis, ∗*p* < 0.05.

In COPSAC_2010_, undetectable maternal 12-HETE levels during pregnancy compared to detectable levels was significantly associated with an increased risk of asthma in offspring from birth to 10 years in a Cox regression analysis of age at onset adjusted for the n-3 LCPUFA RCT, vitamin D RCT, and gender (aHR = 1.62; 95% confidence interval, 1.11–2.35; *p* = 0.013) (Figure 1A); the association was similar for risk of asthma from birth to 6 years (aHR = 1.50; 0.45–1.00; *p* = 0.048). In VDAART, undetectable maternal 12-HETE levels during pregnancy was not associated with the risk of asthma/recurrent wheeze in offspring from birth to 6 years but showed same direction of association (aHR = 1.21; 0.87–1.69; *p* = 0.253) (Figure 1B). When analyzing early-life asthma/wheeze diagnosed at age 3 years in a meta-analysis across the two cohorts, a significant association was observed (aOR = 1.44; 1.01–2.06; *p* = 0.04) (Figure 1C). Further, the daily symptom diary data from COPSAC_2010_ suggested an early-life association, as undetectable compared to detectable maternal pregnancy. 12-HETE levels showed more episodes with asthma-like symptoms (i.e., cough, wheeze and/or breathlessness) in the child’s third year of life in a quasi-Poisson regression analysis (aIRR = 1.39; 1.11–1.76; *p* = 0.005) (Figure 1D). Overall, these results suggest an association between maternal 12-HETE level during pregnancy and asthma symptoms during early life in both cohorts.

Thereafter, we explored early childhood respiratory morbidity by investigating risk of upper and lower airway infections during the first 3 years of life. Using quasi-Poisson regression to analyze the number of infection episodes that was available in both COPSAC_2010_ and VDAART, a meta-analysis of the cohorts showed that undetectable maternal pregnancy 12-HETE was significantly associated with an increased number of colds at age 0–1 year (aIRR = 1.09; 1.01–1.18; *p* = 0.03), age 1–2 years (aIRR = 1.12; 1.02–1.23; *p* = 0.01), and age 0–3 years (aIRR = 1.10; 1.03–1.19; *p* = 0.005) (Figure 1C). There were no associations with age at onset or the number of pneumonia, croup, acute tonsillitis, or acute otitis media (Figure S1; Table S4).

### Maternal pregnancy 12-HETE levels and infant airway microbiota

**Figure 2:**
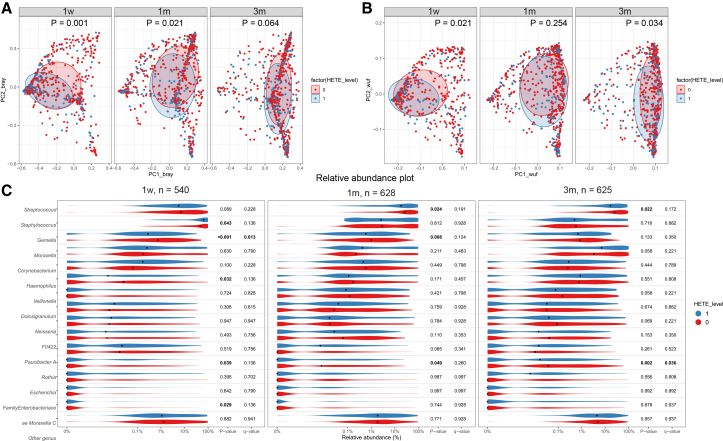
Maternal 12-HETE and infant airway microbiota (A) Association between maternal pregnancy 12-HETE levels and airway microbiota β-diversity (Bray-Curtis distance) at 1 week, 1 month, and 3 months after birth. (B) Association between maternal pregnancy 12-HETE levels and infant airway microbiota β-diversity (weighted unifrac distance) at 1 week, 1 month, and 3 months after birth. (C) Association between maternal pregnancy 12-HETE levels and airway microbiota relative abundances at 1 week, 1 month, and 3 months after birth among the 15 most abundant genera. (1, detectable 12-HETE levels; 0, undetectable 12-HETE levels).

We investigated potential drivers of the association between maternal pregnancy 12-HETE and offspring respiratory morbidity focusing on the infant airway microbiota composition, because recent studies have emphasized a role of 12-HETE in AM self-renewal and maintenance during lung development in mice.^10^ As AMs serve as the primary defense line in the lower airways,^29^ 12-HETE could potentially influence the airway microbiota structure. Data for this analysis were only available in COPSAC_2010_, where we observed associations between maternal pregnancy 12-HETE levels and the overall airway microbiota composition in the offspring (β-diversity by weighted unifrac or Bray-Curtis), with the largest association at the earliest age with attenuating strength for 1- and 3-month airway microbiota at 1 week (Bray-Curtis distance: F = 3.5, R^2^ = 0.5%, *p* = 0.001; weighted unifrac distance: F = 3.06, R^2^ = 0.5%, *p* = 0.021), 1 month (Bray-Curtis distance: F = 2.1, R^2^ = 0.3%, *p* = 0.021; weighted unifrac distance: F = 1.27, R^2^ = 0.2%, *p* = 0.254), and 3 months (Bray-Curtis distance: F = 1.6, R^2^ = 0.2%, *p* = 0.064; weighted unifrac distance: F = 2.39, R^2^ = 0.3%, *p* = 0.034) (Figures 2A and 2B).

Further examination of the relative abundances of the most common bacterial genera showed associations between maternal pregnancy 12-HETE levels and infant airway microbiota relative abundance at 1 week, 1 month, and 3 months after birth. Specifically, undetectable maternal pregnancy 12-HETE levels associated with higher abundance of *Streptococcus*, *Gemella*, and *Rothia* (Wilcoxon rank-sum test, *p* < 0.05); the associations with *Gemella* and *Rothia* remained significant after multiple testing correction (false discovery rate [FDR]<0.05), and higher abundance of *Rothia* was found across time points (Figure 2C). In our previous study, we found several groups of microbial genera at 1 month of age, with moderate to high pairwise correlations, and used a cross-validated sparse partial least squares (PLS) model to identify jointly relevant taxa that were used to construct an airway bacterial asthma score, which strongly associated with asthma at age 6 years.^31^ Here, we utilized this age 1 month airway bacterial score and found that undetectable maternal pregnancy 12-HETE levels were associated with a higher airway bacterial asthma score (beta-estimate = 0.14; −0.004 to 0.29; *p* = 0.05). A causal mediation analysis showed that there was no evidence of mediation (average causal mediation effect [ACME], p-ACME = 0.20).

### Maternal pregnancy 12-HETE levels and infant airway immune profiles

**Figure 3:**
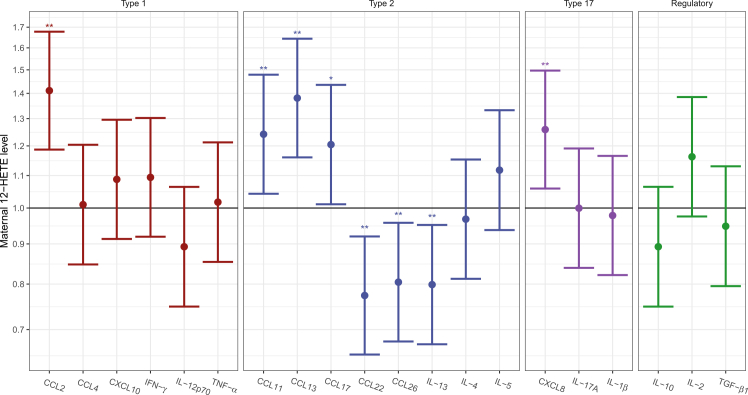
Maternal 12-HETE and airway immune profiles Association between maternal 12-HETE levels and the infant airway immune profile. Linear regression analysis, ∗*p* < 0.05, ∗∗FDR < 0.05.

Subsequently, we explored the association between maternal pregnancy 12-HETE levels and unstimulated *in situ* levels of immune mediators measured in airway mucosal lining fluid collected on filter paper strips at age 1 month in COPSAC_2010_.^31^ The analyses showed that undetectable maternal 12-HETE levels at week 24 of pregnancy were associated with significantly higher levels of CCL2 related to a type 1 immune response pattern; higher CCL11, CCL13, and CCL17 related to a type 2 immune response pattern; higher CXCL8 related to a type 17 immune response pattern; and lower levels of CCL22, CCL26, and interleukin (IL)-13 (Th2) after correction for multiple testing by FDR (Figure 3). The findings were similar after adjusting the analyses for siblings, maternal atopy, and the embedded pregnancy RCTs of n-3 LCPUFA and high-dose vitamin D, except the signal for IL-13, which was no longer significant.

Finally, we investigated an airway asthma immune score generated in our previous study,^31^ which was a score constructed in relation to the bacterial asthma score using a cross-validated sparse PLS as described above. We found that undetectable maternal pregnancy 12-HETE levels were associated with a higher airway asthma immune score: beta-estimate = 0.33; 0.141–0.520; *p* < 0.001. Further, a causal mediation analysis showed that 7% of the effect of maternal 12-HETE on asthma risk at age 0–10 years was mediated through an altered airway asthma immune score at age 1 month (p-ACME = 0.04) (Figure S2).

### Maternal 12-HETE levels and n-3 LCPUFA supplementation during pregnancy

**Figure 4:**
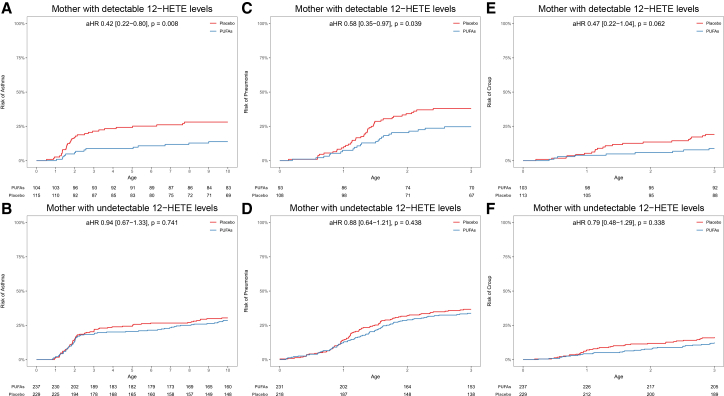
Prenatal n-3 LCPUFA supplementation RCT and offspring respiratory outcomes in COPSAC_2010_ (A and B) Kaplan-Meier curves show association between prenatal n-3 LCPUFA supplementation and risk of childhood asthma in strata of detectable and undetectable maternal 12-HETE levels during pregnancy. (C and D) Kaplan-Meier curves show association between prenatal n-3 LCPUFA supplementation and risk of pneumonia in strata of detectable and undetectable maternal 12-HETE levels during pregnancy. (E and F) Association between prenatal n-3 LCPUFA supplementation and risk of croup in strata of detectable and undetectable maternal 12-HETE levels during pregnancy.

In the COPSAC_2010_ RCT, we previously showed that prenatal n-3 LCPUFA supplementation significantly reduced the risk of wheeze and asthma in offspring by 32%.^45^ Further, a mechanistic study has suggested that n-3 LCPUFA supplementation promotes the polarization of macrophages into an M2 phenotype through GPR120, which is associated with anti-inflammatory effects.^43^^,^^44^ Building on these findings, we investigated whether the impact of prenatal n-3 LCPUFA supplementation on risk of childhood asthma development depended on maternal 12-HETE levels. The analyses showed that among mothers with detectable 12-HETE levels, prenatal n-3 LCPUFA supplementation during pregnancy significantly reduced the risk of childhood asthma by 58% compared to placebo (aHR = 0.42; 0.22–0.86; *p* = 0.008), whereas there was no effect of n-3 LCPUFA among mothers with undetectable 12-HETE levels (aHR = 0.94, 0.67–1.33; *p* = 0.741). This interaction between maternal 12-HETE levels and n-3 LCPUFA supplementation on risk of childhood asthma was statistically significant (p-interaction = 0.039) (Figures 4 and S3). This suggests that maternal 12-HETE levels may play a critical role in determining the effectiveness of n-3 LCPUFA supplementation during pregnancy, aligning with previous animal studies indicating that 12-HETE influences AM levels in the lungs,^10^ which may be crucial for immune maturation and respiratory morbidity.^13^

In addition to asthma, we also examined the effect of prenatal n-3 LCPUFA supplementation on risk of respiratory infections, as our previous studies have shown a protective effect on both lower respiratory tract infections (pneumonia)^45^ and upper airway infections (croup).^48^ For both pneumonia and croup, we found a pattern similar to asthma risk, showing that among mothers with detectable 12-HETE levels, the prenatal n-3 LCPUFA supplementation was associated with a significantly reduced risk at age 0–3 years: pneumonia, aHR = 0.58, 0.35–0.97; *p* = 0.039, and croup, aHR = 0.47; 0.22–1.04; *p* = 0.062, whereas there was no association among mothers with undetectable 12-HETE levels; however, there was no significant interaction between maternal 12-HETE levels and n-3 LCPUFA supplementation (p-interactions = 0.187) (Figures 4 and S3). The n-3 LCPUFA supplementation showed no reduced risk of other upper airway infections, including colds, acute tonsillitis, or acute otitis media, in analyses stratified by maternal 12-HETE levels (Figure S4). Overall, these findings suggest that the protective effects of prenatal n-3 LCPUFA supplementation on childhood asthma and certain specific respiratory infections are influenced by maternal 12-HETE levels.

### Maternal 12-HETE levels and n-3 LCPUFA intake during pregnancy

**Figure 5:**
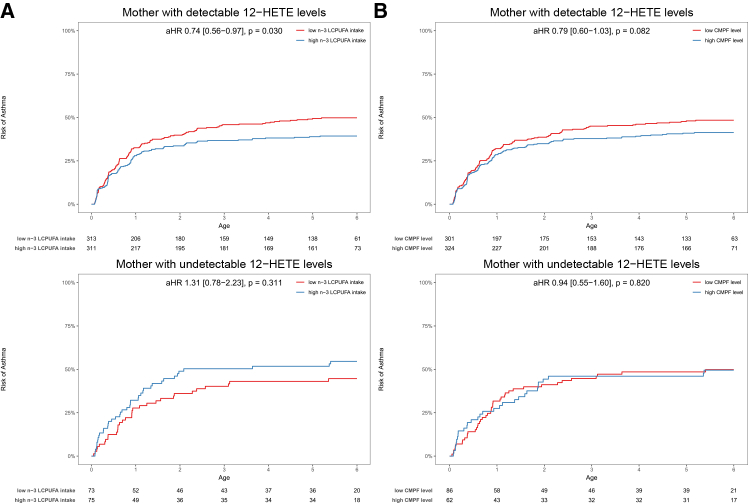
Prenatal n-3 LCPUFA intake and offspring respiratory outcomes in VDAART (A) Kaplan-Meier curves show association between FFQ estimated dietary n-3 LCPUFA intake during pregnancy and risk of childhood asthma stratified by maternal 12-HETE levels in VDAART. (B) Kaplan-Meier curves show association between maternal CMPF levels and risk of childhood asthma stratified by maternal 12-HETE levels strata in VDAART.

In the VDAART cohort, the absence of a prenatal n-3 LCPUFA supplementation RCT led us to analyze maternal dietary habits during pregnancy using a westernized diet score derived from a Food Frequency Questionnaire (FFQ), to approximate prenatal fish oil and fatty fish intake, i.e., n-3 LCPUFA, where a lower westernized diet score correlated to higher intake. Similar to the findings from the COPSAC_2010_ n-3 LCPUFA RCT, we observed a significant interaction between maternal 12-HETE levels and the westernized diet score estimated dietary n-3 LCPUFA intake in relation to offspring asthma risk (p-interaction = 0.028) in VDAART. Specifically, among mothers with detectable 12-HETE levels, a high n-3 LCPUFA intake during pregnancy, i.e., a score below the median, was associated with a reduced risk of childhood asthma from birth to 6 years (aHR = 0.74; 0.56–0.97; *p* = 0.03), whereas there was no association among mothers with undetectable 12-HETE levels (aHR = 1.31; 0.78–2.23; *p* = 0.30) (Figure 5A).

We also assessed the impact of n-3 LCPUFA intake during pregnancy on early-life respiratory infections using the westernized diet scores. Among mothers with detectable 12-HETE levels, high vs. low dietary n-3 LCPUFA intake was associated with a lower risk of pneumonia (aIRR = 0.61; 0.41–0.89; *p* = 0.01) and acute otitis media (aIRR = 0.90; 0.81–1.0; *p* = 0.06) from birth to 3 years (Table S5), but there was no significant interaction between maternal 12-HETE levels and dietary n-3 LCPUFA intake for any of the respiratory infection outcomes similar to the findings observed in COPSAC_2010_.

To further validate our findings on prenatal n-3 LCPUFA exposure in VDAART, we utilized our previous finding that the plasma level of 3-carboxy-4-methyl-5-propyl-2-furan propanoic acid (CMPF) is a strong biomarker of fish oil and n-3 LCPUFA intake during pregnancy.^45^ Similar to the results from the FFQ estimated dietary n-3 LCPUFA intake, we found that among mothers with detectable 12-HETE levels, high maternal CMPF levels, i.e., above the median, showed a trend toward a reduced risk of childhood asthma from birth to 6 years: aHR = 0.79; 0.60–1.03; *p* = 0.082, whereas there was no association among mothers with undetectable levels: aHR = 0.94; 0.55–1.60; *p* = 0.820 (Figure 5B). Interestingly, high maternal CMPF levels were significantly associated with asthma/wheeze in early childhood, i.e., at 3 years: aOR = 0.53; 0.34–0.83; *p* = 0.005, but there was no significant interaction between maternal 12-HETE and CMPF levels for either of these outcomes. Further, only one significant association was observed between maternal CMPF levels and respiratory infections, which was for acute tonsillitis from birth to 3 years among mothers with detectable 12-HETE levels (aIRR = 1.51; 1.11–2.03; *p* = 0.008) (Table S6). These findings in VDAART provide further evidence supporting that the preventive effect of increased prenatal n-3 LCPUFA exposure on risk of childhood asthma depends on maternal 12-HETE levels during pregnancy.

## Discussion

Our study sheds light on the role of maternal 12-HETE during pregnancy in the development of childhood asthma and respiratory infections, suggesting a mechanism of action through an altered infant airway microbiota and airway immune profile. These human data are in line with recent research on the role of 12-HETE in neonatal imprinting of AMs, where undetectable 12-HETE levels have been linked with an elevated risk of respiratory morbidity in mice.^10^

Our analysis revealed that undetectable maternal 12-HETE levels at week 24 of pregnancy were associated with 62% increased risk of childhood asthma from birth to 10 years in the large prospective population-based COPSAC_2010_ mother-child cohort. A recent study in genetically deficient mice (Alox15^−/−^), lacking 12-HETE, has shown that neonatal neutrophil-derived 12-HETE is necessary for the self-renewal and maintenance of AMs during lung development. Despite intact seeding and differentiation of AM progenitors, the absence of 12-HETE resulted in a significant reduction of AMs in these mice.^10^ Our study extends these findings by utilizing maternal plasma metabolomics data from two clinical cohorts to support these findings. In VDAART, which comprises a high-risk and racially diverse population, the direct association between maternal 12-HETE levels and increased risk of childhood asthma until age 6 was not significant. One possible explanation is the timing of metabolomics measurements, where 12-HETE was measured in VDAART at week 32 of pregnancy, i.e., late pregnancy, compared to week 24 in COPSAC_2010_, i.e., mid-pregnancy. Given that 12-HETE plays a pivotal role during early lung development, this later measurement may have diminished the ability to detect its influence on asthma risk at age 6 years. Another possible explanation involves a dual-protection mechanism observed across both cohorts. Specifically, detectable 12-HETE levels were associated with a decreased risk of asthma and infections only among mothers with higher n-3 LCPUFA intake. These results provide a possible prevention strategy; both adequate 12-HETE levels and sufficient n-3 LCPUFA intake may be important for optimal respiratory health and reduced risk of infections and asthma. Nevertheless, a meta-analysis across the two cohorts showed a significant association between undetectable 12-HETE levels in pregnancy and increased risk of asthma/wheeze at 3 years of age, indicating that 12-HETE predominantly exerts its protective effects on asthma/wheeze in early childhood.

Studies have shown that asthma patients often experience a skewing of macrophages toward a pro-inflammatory (M1) phenotype or a dysregulation in the balance between pro-inflammatory (M1) and anti-inflammatory (M2) macrophages, which contributes to asthma pathogenesis.^34^ Our human clinical data showed that maternal 12-HETE levels were associated with long-term effects on development of asthma until age 10 years, which support the hypothesis that the colonization and proper functioning of AMs during early life can have long-term consequences on lung health. However, we did not assess neonatal AMs in our study and cannot infer causality. Future work, including targeted studies in animal models and human neonatal samples, will be needed to test this mechanism directly.

Moreover, impaired AM phagocytosis and increased apoptosis have been observed in children with poorly controlled asthma, possibly explaining the increased prevalence and severity of lower respiratory tract infections in these individuals.^17^ To investigate this, we analyzed the association between maternal 12-HETE levels and early-life respiratory infections. We found that undetectable maternal 12-HETE levels were associated with increased risk of colds and showed a trend of more episodes with asthma-like symptoms in the first 3 years of life in COPSAC_2010_. In VDAART, we similarly observed an increased number of colds in the first year of life, and meta-analyses of the two cohorts revealed a significant association between undetectable maternal 12-HETE levels and increased risk of colds during the first 3 years of life, particularly driven by more colds in the first year of life. These findings provide further evidence that insufficient 12-HETE levels may predispose children to increased risk of early-life respiratory infections.

Overall, these analyses of the association between maternal 12-HETE and offspring respiratory diseases support the hypothesis that undetectable 12-HETE levels during pregnancy may result in incomplete AM seeding and dysfunction, thereby increasing the risk of childhood asthma and respiratory infections. Thereafter, we explored underlying mechanisms and found that maternal 12-HETE levels during pregnancy were associated with the infant airway microbiota structure at 1 week, 1 month, and 3 months after birth. Particularly, undetectable maternal 12-HETE levels were associated with higher abundance of *Streptococcus*, *Gemella*, and *Rothia* at all three time points, and with higher abundance of *Haemophilus influenzae* and *Moraxella catarrhalis* at 1 week. Interestingly, previous studies have demonstrated that neonatal bacterial airway colonization at 1 month with *Streptococcus pneumoniae*, *Haemophilus influenzae*, and/or *Moraxella catarrhalis* was associated with an increased risk of asthma until 5 years in COPSAC_2000_,^30^^,^^49^ as well as asthma and allergy endpoints until age 7 years.^33^ Additionally, the composition of the airway microbiota at 1 month has also previously been linked with risk of asthma until age 6 years in COPSAC_2010_.^31^ Here, we expand on those findings by showing that undetectable maternal 12-HETE levels were associated with a higher airway bacterial asthma score, which partially mediated 12-HETE’s association with asthma at age 10. Thus, our study provides evidence suggesting that maternal 12-HETE during pregnancy may influence the composition of the infant airway microbiota, which could be targeted to ensure a healthy development of the infant airway microbiota and protect against development of childhood asthma.

Previous studies in COPSAC_2010_ have shown that a higher relative abundance of these airway bacteria at age 1 month was associated with an airway immune profile dominated by reduced tumor necrosis factor alpha and IL-1β levels and increased CCL2, CCL11, CCL13, and CCL17 levels, which was associated with risk of asthma.^31^ This suggests a mechanism of topical airway microbiota-immune interactions in early infancy that predisposes to childhood asthma. In the current study, we found that undetectable maternal 12-HETE levels were associated with higher airway levels of CCL2, CCL11, CCL12, CCL17, and CXCL8, as well as lower levels of CCL22, CCL26, and IL-13, underscoring the role of airway microbiota-immune interactions in mediating the relationship between maternal 12-HETE levels and risk of childhood asthma. In addition, we found that undetectable maternal 12-HETE levels were associated with a higher airway immune asthma score, which was also associated with an increased risk of asthma at age 10. Further, mediation analysis revealed that the effect of maternal 12-HETE levels on asthma risk from birth to age 10 was partly mediated through alterations in the airway immune score at age 1 month. Interestingly, CCL2 is crucial for recruiting monocytes and macrophages to the site of inflammation in the airway and is further related to Th2-type inflammation.^50^^,^^51^ Elevated levels of CCL2 in the airway epithelium may drive the recruitment of AMs, which play a significant role in regulating airway inflammation and remodeling in allergic asthma.^50^ One hypothesis is that insufficient AM colonization leads to higher airway levels of CCL2 as a compensatory mechanism to attract additional AMs. This hypothesis is supported by our finding that elevated CCL2 levels were associated with undetectable 12-HETE and an increased asthma risk. These findings suggest an important role of 12-HETE in influencing the early airway microbiota structure and training of the airway immune profile, which subsequently may impact asthma risk in childhood. Further, undetectable levels of maternal 12-HETE were linked to an imbalance in the expression of key airway chemokines and cytokines, characterized by elevated levels of early-phase inflammatory markers such as CCL11, CCL12, and CCL17, which are known to recruit eosinophils and Th2 cells, driving allergic inflammation.^49^^,^^50^^,^^51^ In contrast, regulatory markers involved in the resolution of inflammation, including CCL22, CCL26, and IL-13,^52^^,^^53^^,^^54^ were significantly reduced. This suggests that in the absence of 12-HETE, the topical airway immune system is skewed toward an overactive early inflammatory response, while lacking the regulatory signals necessary to control and resolve inflammation. This imbalance may contribute to the development of chronic airway inflammation and increase the susceptibility to respiratory infections and asthma in later life.^52^ These findings underscore the dynamic nature of immune regulation, where early-life disruptions in lipid mediator signaling, such as the absence of 12-HETE, can have lasting consequences on lung health.

Last, we explored whether levels of maternal 12-HETE affected the preventive potential of prenatal n-3 LCPUFA supplementation on childhood asthma.^45^ This exploration was driven by a previous identification of GPR120 as a receptor for n-3 fatty acids on macrophages, suggesting a mechanism for the observed anti-inflammatory effects of n-3 LCPUFA by promoting macrophages into an M2 phenotype. The composition of gut bacteria determines the effectiveness and physiological outcomes of consuming n-3 LCPUFA.^53^ Research also shows that 12-HETE plays a crucial role in the gut microbiome’s effects on host metabolism and inflammation and signaling via mu-opioid receptors and PPARγ.^54^ Prebiotics like fructooligosaccharides increase 12-HETE production, linking gut microbiota modulation to improved metabolic health and reduced inflammation through this specific lipid mediator.^55^ Notably, we observed that prenatal n-3 LCPUFA supplementation compared to placebo conferred protective effects against childhood asthma only among children born to mothers with detectable levels of 12-HETE, which may suggest that optimal AM colonization during early development might enhance the efficacy of such supplementation strategy. In the context of incomplete AM colonization, maternal 12-HETE is crucial for the proper development and function of AMs; i.e., if AMs are not adequately colonized or functionally impaired, the anti-inflammatory benefits of n-3 LCPUFA supplementation may be compromised. At baseline, higher levels of EPA, DHA, and AA at week 24 associated with higher levels of 12-HETE, which is biologically plausible because 12-HETE is an eicosanoid lipid mediator formed from AA via LOX activity. After adjustment for baseline LCPUFA levels, the association between undetectable 12-HETE levels and asthma to age 10 persisted. In our previous study, the prenatal n-3 LCPUFA RCT reduced asthma risk up to age 5, particularly among children whose mothers had lower baseline EPA+DHA levels. This pattern is consistent with our current finding that the RCT effect is most pronounced among pregnancies with undetectable maternal 12-HETE levels. While we did not observe a direct association between baseline EPA+DHA levels and offspring asthma, maternal 12-HETE was significantly related to asthma risk through age 10. Given that 12-HETE is a bioactive eicosanoid produced from AA by LOX, these findings suggest that the downstream mediator 12-HETE functions as a biomarker of maternal lipid-inflammatory status linked to offspring asthma risk and may condition responsiveness to prenatal n-3 LCPUFA. Our results in COPSAC_2010_ were validated in the independent VDAART cohort, where higher n-3 LCPUFA dietary intake, approximated by an FFQ score and by the plasma biomarker CMPF, was associated with a reduced risk of childhood asthma only among children born to mothers with detectable levels of 12-HETE. These findings suggest a potential link between anti-inflammatory effects of fish oil and fatty fish intake in pregnancy and colonization of AMs facilitated by maternal 12-HETE. We found a similar pattern of reduced risk of respiratory infections only among children born to mothers with detectable 12-HETE in pregnancy compared to no effect among children born to mothers with undetectable 12-HETE, but without significant interaction. Previous studies have already shown that FADS gene variants can modify the protective effect of prenatal and early-life n-3 LCPUFA intake against childhood asthma, but this study proposes additional mechanisms as maternal FADS variants were unrelated to 12-HETE levels. 12-HETE, derived from omega-6 unsaturated fatty acids such as AA, is produced through the action of LOX enzymes, specifically ALOX12 and ALOX15. In our data, we did not observe correlations between environmental factors during the week 24 of pregnancy and 12-HETE levels. A limitation of this study is that we currently did not observe what specifically determines the levels of 12-HETE during pregnancy. However, we propose that this molecule, as a downstream product of omega-6 unsaturated fatty acid metabolism, may provide health benefits.^56^^,^^57^ Thus, our study provides a perspective, emphasizing that the effect of prenatal n-3 LCPUFA intake in reducing asthma risk may also depend on 12-HETE levels during pregnancy.

### Limitations of the study

This study has several strengths. First, the centralized, prospective, and longitudinal clinical follow-up ensures consistent monitoring, data collection, and a nuanced understanding of the temporal aspects of asthma/wheeze development. Second, daily symptom recording from birth minimizes recall bias and provides a comprehensive picture of symptom progression. Third, the use of the COPSAC clinic as the primary health care center establishes a standardized approach to diagnosis and treatment, reducing heterogeneity and improving diagnostic reliability. Additionally, the incorporation of maternal metabolite profiles and the integration of infant airway microbiota and airway immune profiles provide valuable mechanistic insights. Fourth, the utilization of evidence derived from two independent cohorts strengthens the interpretation of findings that were present in both cohorts, enhancing the robustness and generalizability of the results.

However, the study also has its limitations. First, the main limitation of the study is the lack of an n-3 LCPUFA RCT as well as airway microbiota and airway immune profiles in the VDAART cohort. The second limitation is that the racial composition of the two cohorts differs with COPSAC_2010_ predominantly being Caucasian and VDAART being multiracial. Further, COPSAC_2010_ is a population-based cohort, while VDAART is a high-risk cohort. Third, regarding pathway coverage, 12-HETE is an endogenous BLT2 agonist, whereas LTB4 is the high-affinity agonist of BLT1 and can activate BLT2 at higher concentrations; however, LTB4 and related oxylipins (e.g., 5-HETE, 15-HETE, and DHA-derived HDoHEs) were not reported in our HD4 runs and were, therefore, unavailable for analysis. Fourth, we did not collect maternal microbiome data, which precludes testing bidirectional relationships between the maternal microbiome, 12-HETE, and n-3 LCPUFA intake/responsiveness. Fifth, we lacked infant lung tissue or other direct assessments of neonatal AMs; therefore, we cannot determine causality or directionality between maternal 12-HETE and neonatal AM biology. Finally, this study was exploratory and not predefined in the analyses of the n-3 LCPUFA intervention.

### Conclusion

This study shows a significant association between maternal 12-HETE levels in pregnancy and risk of childhood asthma and early-life respiratory infections and suggests that the association is explained by alterations of the infant airway microbiota composition and airway immune profiles. Further, we found that maternal 12-HETE levels in pregnancy determined the protective effect on childhood asthma of a prenatal n-3 LCPUFA RCT and prenatal n-3 LCPUFA exposure in an observational replication cohort. Monitoring and potentially modifying maternal 12-HETE levels during pregnancy could be a valuable approach in reducing the incidence of childhood asthma and respiratory infections.

## Resource availability

### Lead contact

Further information and requests for resources and reagents should be directed to and will be fulfilled by the lead contact, Professor Bo Chawes (chawes@copsac.com).

### Materials availability

This study did not generate new unique reagents.

### Data and code availability

• The 16S rRNA gene sequencing data are deposited at the Sequence Read Archive (SRA) with the accession no. PRJNA340273.
• Individual-level personally identifiable clinical data from the children participating in the cohort cannot be made publicly available, to protect the privacy of the participants and their families, in accordance with the Danish Data Protection Act and European Regulation 2016/679 of the European Parliament and of the Council (GDPR), which prohibit distribution even in pseudo-anonymized form. However, research collaborations are welcome, and data can be made available under a joint research collaboration by contacting the COPSAC Data Protection Officer (DPO), Ulrik Ralfkiaer, PhD (administration@dbac.dk). Requests will be answered within 2 weeks. Data use is restricted to purposes within childhood health and disease.
• This paper does not report original code.
• Any additional information required to reanalyze the data reported in this paper is available from the lead contact upon request.

## Acknowledgments

Access to data and data analysis: B.C. had full access to all the data in the study and took responsibility for the integrity of the data and the accuracy of the data analysis. Funding/support: COPSAC (Copenhagen Prospective Studies on Asthma in Childhood) is funded by private and public research funds, which are all listed on www.copsac.com. 10.13039/501100003554The Lundbeck Foundation, Danish State Budget, 10.13039/100007398Danish Council for Strategic Research, 10.13039/501100004836Danish Council for Independent Research, and The Capital Region Research Foundation have provided core support for COPSAC. The study is further supported by the following 10.13039/100000002NIH grants: R01HL129735, R01HL129735, R01HL123915, U19AI168643, and R01HL141826, and UH3OD023268. C.E.W. acknowledges support from the 10.13039/501100003793Swedish Heart-Lung Foundation (HLF 20230463, HLF 20210519) and the 10.13039/501100004359Swedish Research Council (2022-00796). This project has received funding from the European Research Council under the European Union’s Horizon 2020 research and innovation program (grant agreement no. 946228). N.B. was funded by 10.13039/501100003554The Lundbeck Foundation (R381–2021-1428). Role of the funder/sponsor statement: The funding sources had no role in the design and conduct of the study; collection, management, analysis, and interpretation of the data; preparation, review, or approval of the manuscript; and decision to submit the manuscript for publication. Additional contributions: We thank the children and families of the COPSAC cohort and the VDAART cohort for their commitment and invaluable contributions. We also extend our appreciation to the dedicated research teams at both COPSAC and VDAART for their efforts and support throughout this work.

## Author contributions

L.C. conceived the study; co-designed the methodology with B.C.; led formal analysis, data curation, and visualization; and wrote the original draft; N.B., J.T., and J.S. contributed to methodology, investigation, formal analysis, and data curation and co-wrote and revised the manuscript; C.E.W., J.L.-S., K.B., J.S., and B.C. provided resources and obtained funding; B.C. supervised the study and managed project administration; all authors reviewed and edited the manuscript for important intellectual content.

## Declaration of interests

J.L.-S. is a scientific advisor to Precision Inc. and TruDiagnostic Inc. J.T. reports a speaking fee from AstraZeneca. S.T.W. receives royalties from UpToDate and is on the Board of Histolix, a digital pathology company.

## STAR★Methods

### Key resources table

**Table:** 

REAGENT or RESOURCE	SOURCE	IDENTIFIER
**Biological samples**		

Participant blood	The Copenhagen Prospective Studies on Asthma in Childhood 2010 (COPSAC_2010_)	Bisgaard et al.^45^ Bisgaard et al.^58^
Participant blood	The Vitamin D Antenatal Asthma Reduction Trial (VDAART)	Litonjua et al.^59^ Litonjua et al.^60^ Litonjua et al.^61^
Hypopharyngeal samples (Airway micromatem biota)	The Copenhagen Prospective Studies on Asthma in Childhood 2010 (COPSAC_2010_)	Mortensen et al.^62^
Airway mucosal lining fluid	The Copenhagen Prospective Studies on Asthma in Childhood 2010 (COPSAC_2010_)	Følsgaard et al.^63^

**Deposited data**		

The 16S-rRNA sequencing data	The Copenhagen Prospective Studies on Asthma in Childhood 2010 (COPSAC_2010_)	Sequence Read Archive (SRA): PRJNA340273

**Software and algorithms**		

R statistical software (version 4.2.2)	R Core Team	https://www.R-project.org/
BioRender	BioRender	https://www.biorender.com/

### Experimental model and study participant details

#### Study population

The COPSAC_2010_ cohort is a population-based mother–child cohort of 738 pregnant women and their 700 children. The children underwent scheduled and acute care visits until age 10 years for close clinical phenotyping done exclusively by the COPSAC pediatricians. The study physicians were responsible for all diagnoses and treatment of asthma, allergy, and atopic dermatitis, adhering to predefined algorithms.^4^^,^^26^^,^^31^^,^^32^^,^^33^^,^^34^

The VDAART mother–child cohort consists of 881 women with a family history of asthma, eczema, or allergic rhinitis and their 810 children. The children were monitored by telephone every 3 months and in-person annually for 6 years, during which offspring health, respiratory symptoms, and medications were assessed. The mothers participated in an RCT of high-dose vs. standard-dose of vitamin D supplementation during pregnancy.^39^^,^^40^^,^^41^

#### The n−3 LCPUFA RCT

In COPSAC_2010_, pregnant women between 22 and 26 weeks of gestation were enrolled and randomly assigned in a 1:1 ratio to receive either 2.4 g per day of *n*-3 LCPUFA (comprising 55% EPA and 37% DHA) in triacylglycerol form (Incromega TG33/22, Croda Health Care) or placebo (olive oil, containing 72% *n*-9 oleic acid and 12% *n*-6 linoleic acid [Pharma-Tech A/S]). A subset of the mothers (*N* = 623) also participated in a high-dose vs. standard dose of vitamin D RCT in a 2 × 2 factorial design with the *n*-3 LCPUFA intervention.^45^^,^^64^ Both supplements continued until 1 week after delivery, with both investigators and participants blinded to group assignments until the youngest child in the trial reached 3 years of age. The children were followed with scheduled visits at 1 week, 1, 3, 6, 12, 18, 24, 30, 36 months, 6, 8, and 10 years.^58^

#### Data collection

##### Primary clinical endpoint: COPSAC_2010_

Wheeze/asthma from birth to 10 years:

Recurrent wheeze: The diagnosis required minimum five episodes of diary-recorded and physician-verified troublesome lung symptoms lasting at least three consecutive days within the preceding 6 months or 4 weeks of consecutive symptoms.^24^^,^^45^^,^^58^

Persistent wheeze/asthma: The diagnosis required 1) recurrent wheeze, 2) typical asthma symptoms, 3) the need for short-acting inhaled beta-2-agonist, and 4) response to a 3-month trial of inhaled corticosteroids with relapse upon cessation. The diagnosis of “persistent wheeze” was utilized until the age of 3 years, after which the term “asthma” was adopted.^24^^,^^45^^,^^58^

##### Secondary clinical endpoints: COPSAC_2010_

Lower respiratory tract infections: Pneumonia at age 0–3 years was diagnosed in children with significant cough, tachypnea, fever, and abnormal lung auscultation. The diagnosis of pneumonia was based on diary entries.^65^^,^^66^^,^^67^

Upper respiratory tract infections: These included episodes of common cold, acute tonsillitis, croup, and acute otitis media until age 3 years, croup was doctor-diagnosed, while the other infections were documented based on diary entries.^65^^,^^66^^,^^67^

Asthma-like symptoms; These were defined as cough, wheeze, and/or shortness of breath severely affecting the well-being of the child, which were captured in the diaries. An episode was defined as lasting at least 3 consecutive days with a gap of at least 3 symptom-free days between 2 asthma-like episodes.^65^^,^^66^^,^^67^

##### Primary clinical endpoint: VDAART

Recurrent wheeze/asthma from birth to 6 years: Asthma was defined according to the composite of (1) asthma (parental report of physician-diagnosed asthma) and (2) recurrent wheeze (a parental report of recurrent wheeze in the child’s first three years of life) as detailed previously.^59^^,^^60^^,^^61^

##### Secondary clinical endpoints: VDAART

Lower respiratory tract infections: These were defined as parental reports of physician-diagnosed bronchitis, bronchiolitis, or pneumonia. Questions about the presence of these diagnoses were asked every 3 months, allowing for recurrent events. Counts of reported infections were assembled from all available follow-up data on each child.^59^^,^^60^^,^^61^

Upper respiratory tract infections included episodes of common cold, acute tonsillitis, croup, and acute otitis media until age 3 years.^59^^,^^60^^,^^61^

### Method details

#### Maternal metabolomics profile in COPSAC_2010_ and VDAART

Maternal blood samples were taken at week 24 of pregnancy in COPSAC and week 32 of pregnancy in VDAART. An untargeted metabolomic analysis of the plasma samples was performed by Metabolon, Inc. (NC, USA) in both cohorts as previously published.^46^^,^^68^ We specifically investigated levels of maternal 12-HETE in these metabolomic profiles due to its highlighted role in recent research, emphasizing the importance of 12-HETE in neonatal imprinting of AMs. In addition, because of the lack of an *n*-3 LCPUFA RCT in VDAART, we utilized the plasma CMPF level as a biomarker of fish oil and fatty fish intake in VDAART.

Untargeted semiquantitative plasma profiling of 1,138 annotated metabolites is done using Metabolon Inc. (NC, USA) HD4 platforms: 1) two separated reverse phase UPLC-ESI(+)MS/MS methods; 2) UPLC-(−)MS/MS; 3) HILIC/UPLC-(−)MS/MS.^46^ Raw data is extracted, peak identified, and QC processed. Samples are semi-quantified using area-under-the-curve. Peak identification/assignment is done by automated comparison of the ion feature in the experimental samples to a reference library of authenticated standards or recurrent unknown entities. The identification is based on three matching criteria: retention time, mass accuracy and MS/MS spectra. Oxylipin metabolites including 12-HETE are available from plasma profiles in COPSAC_2010_ (24-week gestation, one-week postpartum, 0.5, 1.5, 6 and 10 years), VDAART (10–18-week and 32-week gestation, 1, 3 and 6 years).

12-HETE was called at high confidence by retention time, accurate mass, and MS/MS spectral match to authenticated library standards and semi-quantified from peak area (AUC) with run-day median scaling. Values reported as “0” by the vendor were treated as below the limit of detection (LOD). This motivated our primary exposure definition: detectable vs. undetectable 12-HETE levels.

#### Airway microbiota in COPSAC_2010_

The airway microbiota was collected from children at 1 week, 1 month and 3 months after birth. We examined the airway microbiota using a 16S rRNA gene amplicon sequencing protocol targeting the V4 region; the detailed methods have previously been published.^31^^,^^62^ For the airway bacterial asthma score developed in our prior study,^31^ we modeled asthma at age 6 years (binary outcome) from genus-level relative abundances at 1 month of age using partial least squares (PLS/sPLS). Genus abundances were log-transformed and z-scored. PLS is a statistical modeling framework specifically well suited for dealing with highly collinear or redundant feature data matrices and excels in situations where the number of features/variables (p) are high or even greater than the number of observations (n). Similar to, for example, principal component analysis (PCA), features are combined to components via loadings on the original variables, but in a supervised manner to maximize covariance with an outcome, which can be univariate or multivariate. Here, we selected the optimum number of input variables using repeated 10-fold cross validation (repeated cv, number = 5) of the area under the curve (AUC) statistic to avoid overfitting. The final model was chosen by the highest median AUC value. The predicted values of left out folds were combined to form a bacterial asthma score. Relative importance per taxon was calculated as the median taxon loadings across folds divided by the sum of these median loadings. The seven taxa with the largest contributions were Veillonella (28.1%), Prevotella (23.7%), Gemella (16.3%), Bacillales (12.3%), Bacilli (12.2%), Streptococcus (5.4%), and Lactobacillus (2.1%).

#### Airway immune profiles in COPSAC_2010_

Unstimulated airway mucosal lining fluid was obtained from the children at age 1 month by insertion of filter paper strips based on a synthetic absorptive matrix (fibrous hydroxylated polyester sheets, Accuwik Ultra (cat no. SPR0730), Pall Life Sciences, Portsmouth, Hampshire, UK) in both nostrils for 2 min, followed by immediate storage at −80°C. For immune profiling, the MesoScale Discovery multiplexed array system (MesoScale Discovery, Gaithersburg, MD, USA) was used to analyze a comprehensive panel of cytokines and chemokines.^63^ This included key immune mediators: IL-12p70, CXCL10 (IP-10), interferon-gamma (IFN-γ), tumor necrosis factor-alpha (TNF-α), CCL4 (MIP-1β), CCL2 (MCP-1), CCL13 (MCP-4), IL-4, IL-5, IL-13, CCL11 (eotaxin-1), CCL26 (eotaxin-3), CCL17 (TARC), CCL22 (MDC), IL-17A, IL-1β, CXCL8 (IL-8), transforming growth factor β1 (TGF-β1), IL-10, and IL-2. The selected panel of biomarkers was chosen *a priori* to represent mediators involved in distinct immune responses: Type 1 (Th1/CD8+/NK cells/innate lymphoid cells (ILC) 1), Type 2 (Th2, eosinophils, ILC2), Type 17 (Th17, neutrophils, ILC3), and regulatory T cell (Treg) responses. For airway asthma immune score, immune mediator concentrations were standardized, total sum normalized per sample, log transformed, and z-scored before further analysis. Missing immune mediator values (1.2%) were imputed using the k-nearest-neighbors algorithm prior to normalization. Airway asthma immune score were analyzed in relation to the airway bacterial asthma score using linear models and cross-validated sparse PLS as described above, adjusted for other bacteria using PCA with four components and we use component 1 as the score, higher score (component 1) means higher levels of CCL2, CCL11, CCL13, CCL17.^31^

#### Food frequency questionnaires and pregnancy dietary patterns in COPSAC_2010_ and VDAART

Because of the lack of an *n*-3 LCPUFA RCT in VDAART, we estimated fish oil and fatty fish intake in pregnancy utilizing a principal component analysis (PCA) on responses from Food Frequency Questionnaires (FFQs) in COPSAC_2010_, integrating estimates of energy, macronutrient, and micronutrient intake. The second principal component of the model has been shown to resemble an unhealthy westernized diet FFQ score,^69^ had positive associations with intakes of animal fats, refined grains and high-energy drinks, and negative associations with intakes of fruit, fish, and vegetables, which was employed as the response variable for selecting metabolites associated with a westernized diet. This selection was carried out using a sparse PLS regression model with cross-validated predictions (repeated cv, number = 5, repeats = 10) implemented in the caret package (v6.0.90) in R. Following the identification of representative metabolites, westernized diet metabolome scores were computed for pregnancy week 24 in the COPSAC_2010_ cohort. In the VDAART cohort, which includes metabolome profiling of mothers at week 32 of pregnancy, models trained on the COPSAC_2010_ cohort, with overlapping metabolites, were employed to calculate westernized diet scores mimicking intake of *n*-3 LCPUFA.

### Quantification and statistical analysis

Our data analysis aimed to investigate the relationship between maternal 12-HETE level during pregnancy and respiratory outcomes in children, including asthma and respiratory tract infections in COPSAC_2010_ and VDAART. For asthma outcomes, we employed Cox regression and logistic regression models. The Cox regression models were used to assess the risk of asthma from birth to 10 years in COPSAC_2010_ and from birth to 6 years in VDAART modeling age at onset illustrated by Kaplan-Meier curves. Logistic regression was applied for early-life wheeze/asthma endpoints at 3 and 6 years in both cohorts. For respiratory infection outcomes, Cox regression models were used to evaluate time-to-event data for lower respiratory tract infections (e.g., pneumonia) and upper respiratory tract infections (e.g., colds, croup, acute otitis media, acute tonsillitis) until age 3 years in COPSAC_2010_. Quasi-Poisson regression models were used to examine the association between maternal 12-HETE level and the number of infection episodes in both cohorts. All regression models were adjusted for the RCTs and gender. Additionally, we explored the association between maternal 12-HETE levels and the infant airway microbiota composition and airway immune profile in COPSAC_2010_ with the goal of understanding potential underlying mechanisms.

Furthermore, we assessed whether the impact of *n*-3 LCPUFA supplementation during pregnancy on asthma risk from birth to age 10 years depended on maternal 12-HETE level in COPSAC_2010_. This analysis involved Cox regression models with an interaction term. Subsequently, we conducted analyses to evaluate the effect of *n*-3 LCPUFA intervention on asthma risk within different maternal 12-HETE level strata. To validate our findings in VDAART, we utilized dietary intake data from FFQ and plasma levels of CMPF, a metabolite reflecting fish oil intake38. All statistical analyses were conducted using R software (version 4.2.2).

### Additional resources

ClinicalTrials.gov number, NCT00798226. https://clinicaltrials.gov/study/NCT00798226.

## Supplemental information

Document S1. Figures S1–S4 and Tables S1–S6

Document S2. Article plus supplemental information
