# Supplementary material for: Maternal 12-HETE is associated with childhood asthma and the responses to prenatal omega-3 supplementation
Source: Cell Rep Med. 2026 Mar 17;7(3):102689. doi: 10.1016/j.xcrm.2026.102689 (PMC13006429; doi:10.1016/j.xcrm.2026.102689)
Supplement: Document S1. Figures S1–S4 and Tables S1–S6 [file mmc1.pdf]

Cell Reports Medicine, Volume *7*

Supplemental information

Maternal 12-HETE is associated with childhood asthma and the responses to prenatal omega-3 supplementation

Liang Chen, Nicklas Brustad, Jonathan Thorsen, Tingting Wang, Mina Ali, Julie N. Kyvsgaard, Mario Lovric, Parvaneh Ebrahimi, Yang Luo, Casper-Emil T. Pedersen, Nicole Prince, Rachel S. Kelly, Ann-Marie M. Schoos, Nilo Vahman, Morten A. Rasmussen, Susanne Brix, Augusto A. Litonjua, Scott T. Weiss, Craig E. Wheelock, Jessica Lasky-Su, Klaus Bønnelykke, Jakob Stokholm, and Bo Chawes

Supplementary Information

Figure S1 | Association between maternal 12-HETE level and risk of childhood lower respiratory infections (A: pneumonia) and upper respiratory infections (B: croup, C: cold, D: acute tonsillitis, E: acute otitis media) in COPSAC$_{2010}$.

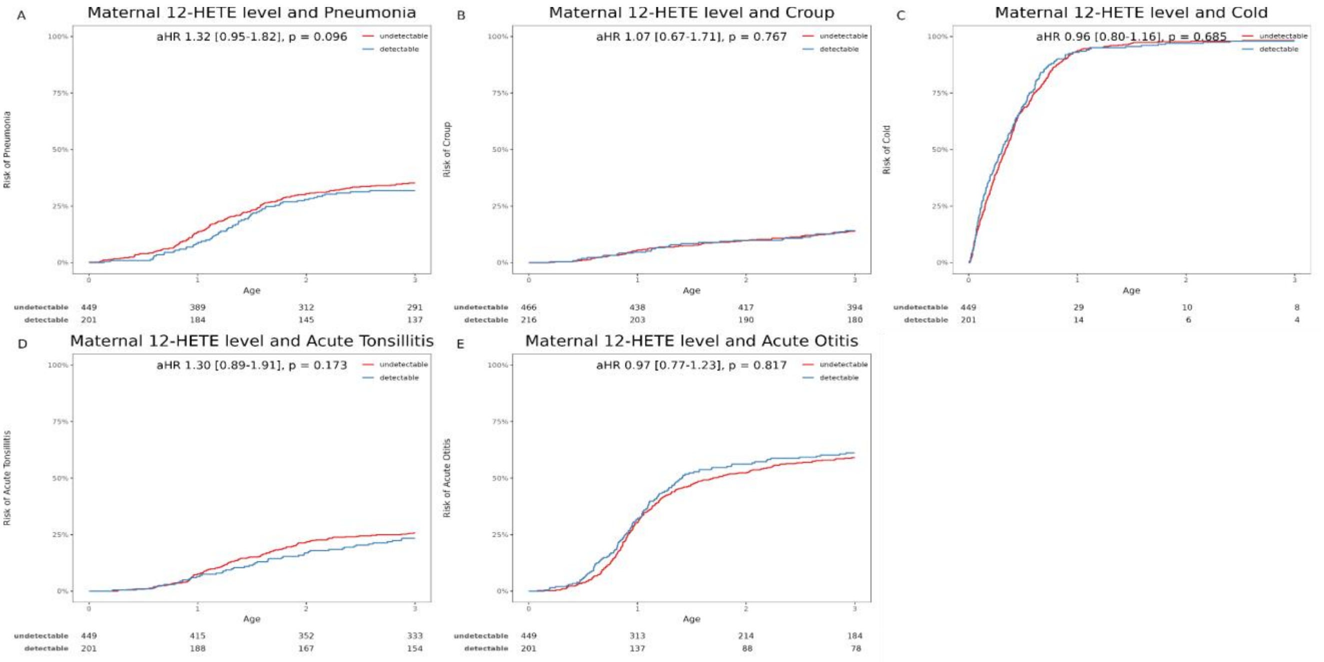

Figure S2 | Mediation analysis of the effect of maternal 12-HETE on asthma risk at age 0-10 years through an altered airway asthma immune score at age 1 month.

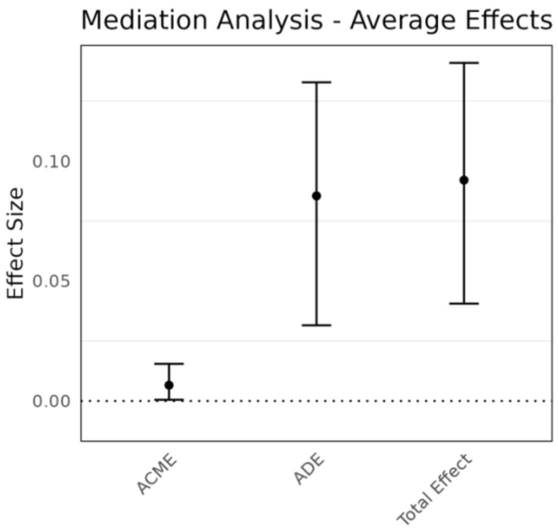

Figure S3 | Association between prenatal 12-HETE levels and risk of childhood asthma, pneumonia and croup in strata of n-3 LCPUFA supplementation and placebo during pregnancy.

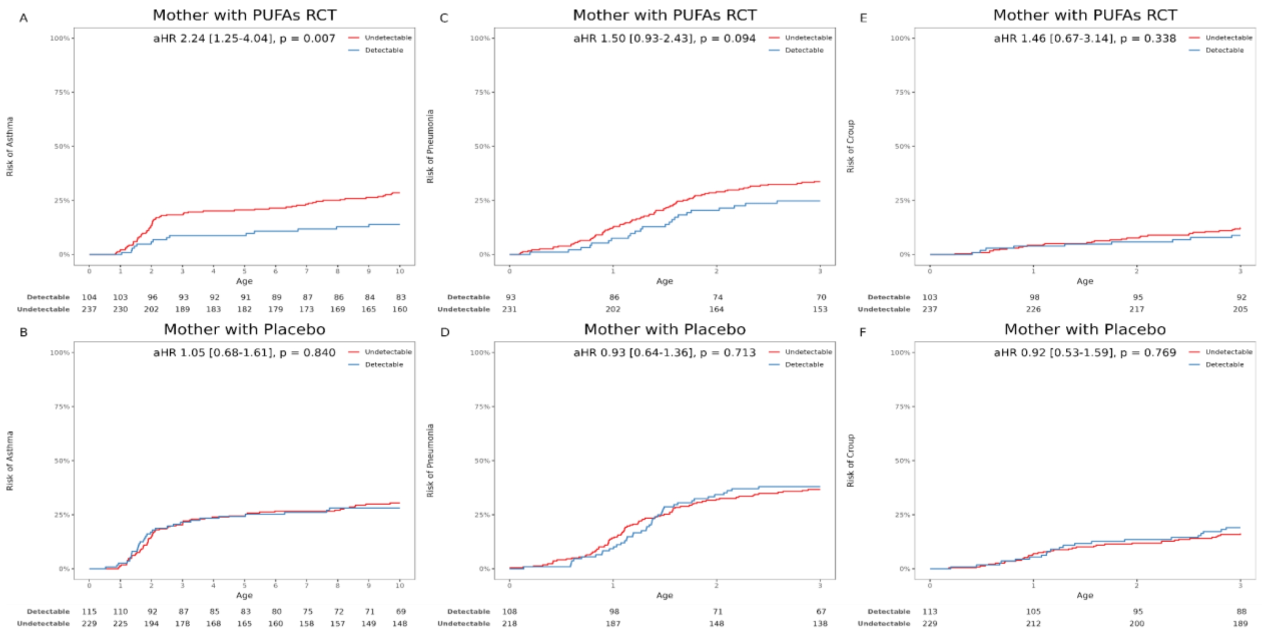

Figure S4 | Association between prenatal n-3 LCPUFA supplementation and risk of childhood upper respiratory infections based on maternal 12-HETE level strata in $COPSAC_{2010}$.

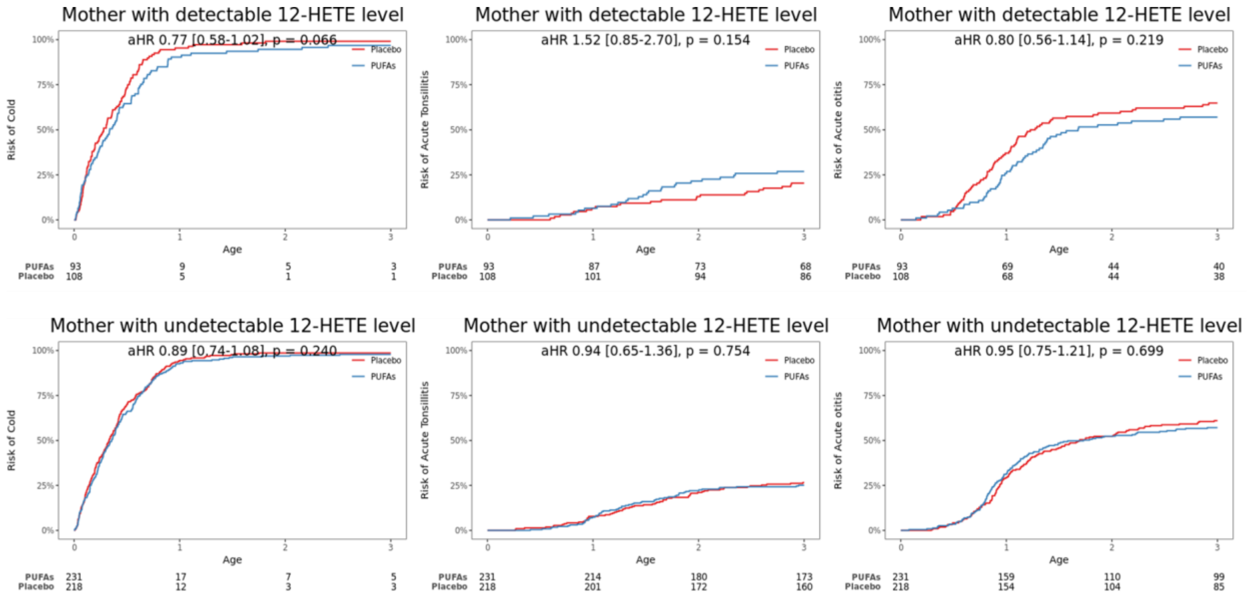

Table S1 | Comparison of baseline characteristics among mothers with detectable vs. undetectable 12-HETE level in COPSAC$_{2010}$.

Characteristic	N	Overall, N = 727[1]	detectable 12-HETE level, N = 236[1]	undetectable 12-HETE level, N = 491[1]	p-value[2]
Mother race	708				0.8
caucasian		678 (96%)	221 (96%)	457 (96%)	
non-caucasian		30 (4.2%)	9 (3.9%)	21 (4.4%)	
Mother income	686				0.6
High		125 (18%)	35 (16%)	90 (19%)	
Low		58 (8.5%)	18 (8.2%)	40 (8.6%)	
Medium		503 (73%)	166 (76%)	337 (72%)	
Mother education level	686				0.055
High		201 (29%)	51 (23%)	150 (32%)	
Low		50 (7.3%)	16 (7.3%)	34 (7.3%)	
Medium		435 (63%)	152 (69%)	283 (61%)	
Mother BMI	708	23.6 (21.5, 26.5)	24.0 (21.8, 27.3)	23.4 (21.5, 26.2)	0.094
Mother FADS Genotype	710				0.5
AA		299 (42%)	104 (45%)	195 (41%)	
AG		318 (45%)	96 (42%)	222 (46%)	
GG		93 (13%)	30 (13%)	63 (13%)	
Mother smoking pregnancy	689				0.6
Non-smoking		665 (97%)	211 (96%)	454 (97%)	
Smoking		24 (3.5%)	9 (4.1%)	15 (3.2%)	
Mother passive smoking pregnancy	689				0.5
Non-passive smoking		589 (85%)	185 (84%)	404 (86%)	
passive smoking		100 (15%)	35 (16%)	65 (14%)	
Antibiotic usage during pregnancy	695	252 (36%)	78 (35%)	174 (37%)	0.6
Mother Asthma	719				0.7
Asthma		193 (27%)	65 (28%)	128 (26%)	
No Asthma		526 (73%)	170 (72%)	356 (74%)	
n-3 LCPUFA RCT	726				0.3
n-3 LCPUFA		360 (50%)	111 (47%)	249 (51%)	
Placebo		366 (50%)	125 (53%)	241 (49%)	
Vitamin D RCT	616				0.054

Placebo	303 (49%)	91 (44%)	212 (52%)
Vitamin D3	313 (51%)	117 (56%)	196 (48%)

[1] n (%); Median (IQR)

[2] Pearson's Chi-squared test; Wilcoxon rank sum test

Table S2 | Comparison of baseline characteristics among mothers with detectable vs. undetectable 12-HETE level in VDAART.

**Characteristic**	**N**	**Overall**, N = 779[1]	**detectable 12-HETE level**, N = 628[1]	**undetectable 12-HETE level**, N = 151[1]	**p-value**[2]
Mother race	779				0.11
American Indian or Alaskan Native		8 (1.0%)	6 (1.0%)	2 (1.3%)	
Asian		34 (4.4%)	28 (4.5%)	6 (4.0%)	
Black or African American		340 (44%)	260 (41%)	80 (53%)	
Native Hawaiian or Other Pacific Islander		11 (1.4%)	9 (1.4%)	2 (1.3%)	
Other		72 (9.2%)	64 (10%)	8 (5.3%)	
White		314 (40%)	261 (42%)	53 (35%)	
Mother BMI	670	27 (23, 32)	27 (23, 32)	26 (22, 31)	0.3
Mother income	772				0.051
High		86 (11%)	70 (11%)	16 (11%)	
Low		327 (42%)	268 (43%)	59 (40%)	
Medium		172 (22%)	147 (24%)	25 (17%)	
NA		187 (24%)	139 (22%)	48 (32%)	
Mother education level	772				0.2
College graduate or Graduate school		262 (34%)	218 (35%)	44 (30%)	
High school, Technical school		229 (30%)	178 (29%)	51 (34%)	
Less than high school		98 (13%)	75 (12%)	23 (16%)	
Some college		183 (24%)	153 (25%)	30 (20%)	
Mother asthma	779				0.7
Asthma		310 (40%)	254 (40%)	56 (37%)	
NA		13 (1.7%)	10 (1.6%)	3 (2.0%)	
No Asthma		456 (59%)	364 (58%)	92 (61%)	
Mother smoking pregnancy	779	18 (2.3%)	14 (2.2%)	4 (2.6%)	0.8
Vitamin D RCT	772				0.2

Placebo	387 (50%)	320 (51%)	67 (45%)
Vitamin D3	385 (50%)	304 (49%)	81 (55%)

[1] n (%); Median (IQR)

[2] Fisher's exact test; Wilcoxon rank sum test; Pearson's Chi-squared test

Table S3 | Comparison of baseline characteristics in COPSAC$_{2010}$ and VDAART.

**Characteristic**	**N**	**Overall**, N = 1,615[1]	**COPSAC2010**, N = 733[1]	**VDAART**, N = 882[1]	**p-value**[2]
Mother race	1,594				<0.001
Other		562 (35%)	31 (4.4%)	531 (60%)	
White		1,032 (65%)	681 (96%)	351 (40%)	
Mother BMI	1,410	24.8 (22.0, 29.1)	23.6 (21.6, 26.5)	26.6 (22.7, 32.0)	<0.001
Mother income	1,496				<0.001
High		218 (15%)	125 (18%)	93 (12%)	
Low		402 (27%)	61 (8.8%)	341 (42%)	
Medium		680 (45%)	504 (73%)	176 (22%)	
NA		196 (13%)	0 (0%)	196 (24%)	
Mother education level	1,496				<0.001
High		666 (45%)	201 (29%)	465 (58%)	
Low		151 (10%)	51 (7.4%)	100 (12%)	
Medium		679 (45%)	438 (63%)	241 (30%)	
Mother Asthma	1,605				<0.001
Asthma		554 (35%)	196 (27%)	358 (41%)	
NA		15 (0.9%)	0 (0%)	15 (1.7%)	
No Asthma		1,036 (65%)	528 (73%)	508 (58%)	
Mother smoking pregnancy	1,534	43 (2.8%)	25 (3.6%)	18 (2.1%)	0.083
Vitamin D RCT	1,424				0.8
Placebo		705 (50%)	304 (49%)	401 (50%)	
Vitamin D3		719 (50%)	314 (51%)	405 (50%)	

[1] n (%); Median (IQR)

[2] Pearson's Chi-squared test; Wilcoxon rank sum test

Table S4 | Association between maternal 12-HETE level and number of respiratory infections from birth to age 3 years (Quasi-Poisson regression) in $COPSAC_{2010}$ and VDAART.

	COPSAC			VDAART			Meta analysis				
Clinical endpoints	**aIRR**	**95%CI**	**P-value**	**aIRR**	**95%CI**	**P-value**	**aIRR**	**95%CI**	**P-value**	**I²**	**Heterogeneity**
Pneumonia 0-1	1.36	0.83-2.36	0.24	1.16	0.47-2.50	0.73	1.30	0.84-2.02	0.24	0.0%	0.74
Pneumonia 1-2	0.89	0.64-1.26	0.50	1.72	0.87-3.21	0.09	1.02	0.76-1.39	0.86	68.4%	0.07
Pneumonia 2-3	1.28	0.77-2.24	0.36	1.72	0.80-3.44	0.14	1.42	0.93-2.18	0.11	0.0%	0.52
Pneumonia 0-3	1.06	0.79-1.45	0.71	1.45	0.84-2.42	0.17	1.15	0.88-1.49	0.31	3.2%	0.31
Cold 0-1	1.05	0.95-1.17	0.33	1.13	1.00-1.27	**0.03**	1.09	1.01-1.18	**0.03**	0.0%	0.38
Cold 1-2	1.16	1.02-1.33	**0.02**	1.08	0.95-1.22	0.21	1.12	1.02-1.23	**0.01**	0.0%	0.42
Cold 2-3	1.24	1.05-1.48	**0.01**	1.02	0.91-1.16	0.65	1.09	0.99-1.21	0.07	67.3%	0.08
Cold 0-3	1.14	1.05-1.48	**0.01**	1.08	0.98-1.19	0.13	1.10	1.03-1.19	**0.005**	0.0%	0.47
Tonsillitis 0-1	0.98	0.52-1.93	0.94	0.80	0.31-1.70	0.59	0.90	0.54-1.50	0.69	0.0%	0.71
Tonsillitis 1-2	1.21	0.76-2.02	0.44	0.60	0.29-1.12	0.13	0.95	0.64-1.41	0.79	63.6%	0.09
Tonsillitis 2-3	0.77	0.41-1.51	0.43	0.63	0.35-1.08	0.12	0.69	0.45-1.06	0.09	0.0%	0.67
Tonsillitis 0-3	1.01	0.68-1.53	0.95	0.65	0.37-1.05	0.10	0.86	0.62-1,18	0.34	43.8%	0.18
Acute otitis media 0-1	0.92	0.68-1.26	0.58	1.09	0.86-1.38	0.46	1.02	0.85-1.24	0.81	0.0%	0.37
Acute otitis media 1-2	0.90	0.69-1.18	0.44	1.13	0.90-1.42	0.28	1.03	0.86-1.23	0.76	39.8%	0.19
Acute otitis media 2-3	1.03	0.70-1.54	0.89	0.98	0.75-1.27	0.91	0.99	0.80-1.24	0.99	0.0%	0.86
Acute otitis media 0-3	0.93	0.75-1.15	0.48	1.05	0.87-1.26	0.59	0.99	0.96-1.15	0.95	0.0%	0.38

Table S5 | Association between FFQ estimated maternal dietary intake of n-3 LCPUFA in pregnancy and respiratory infections in VDAART stratified by maternal 12-HETE level.

**Dietary n-3 LCPUFA intake and Infections in VDAART**						
	**Detectable 12-HETE**			**Undetectable 12-HETE**		
**Clinical endpoints**	**aIRR**	**95%CI**	**P-value**	**aIRR**	**95%CI**	**P-value**
Pneumonia 0-1	0.55	0.29-1.00	0.06	0.91	0.35-2.27	0.84
Pneumonia 1-2	0.69	0.42-1.11	0.14	0.70	0.26-1.72	0.45
Pneumonia 2-3	0.68	0.38-1.15	0.17	1.76	0.81-4.01	0.16
Pneumonia 0-3	0.61	0.41-0.89	**0.01**	1.09	0.55-2.16	0.80
Cold 0-1	1.02	0.96-1.09	0.49	1.00	0.88-1.15	0.95
Cold 1-2	1.02	0.95-1.09	0.65	1.06	0.89-1.25	0.51
Cold 2-3	0.95	0.89-1.01	0.11	0.97	0.83-1.13	0.69
Cold 0-3	0.99	0.94-1.05	0.79	1.00	0.88-1.14	0.99
Tonsillitis 0-1	1.46	0.99-2.12	**0.05**	1.31	0.51-3.49	0.58
Tonsillitis 1-2	1.34	1.02-1.75	**0.03**	1.50	0.74-3.16	0.27
Tonsillitis 2-3	1.15	1.02-1.75	0.32	1.92	1.00-3.88	0.06
Tonsillitis 0-3	1.29	1.02-1.62	**0.03**	1.87	1.02-3.52	**0.05**
Acute otitis media 0-1	0.97	0.85-1.12	0.71	0.89	0.68-1.17	0.42
Acute otitis media 1-2	0.85	0.74-0.98	**0.02**	0.92	0.69-1.22	0.55
Acute otitis media 2-3	0.85	0.73-0.99	**0.04**	0.92	0.69-1.22	0.58
Acute otitis media 0-3	0.90	0.81-1.00	0.06	0.92	0.74-1.13	0.42

Table S6 | Association between CMPF level in pregnancy and respiratory infections in VDAART stratified by maternal 12-HETE level.

**CMPF level and Infections in VDAART**						
	**Detectable 12-HETE**			**Undetectable 12-HETE**		
**Clinical endpoints**	**aIRR**	**95%CI**	**P-value**	**aIRR**	**95%CI**	**P-value**
Pneumonia 0-1	1.52	0.76-2.99	0.23	0.62	1.54-2.29	0.49
Pneumonia 1-2	1.15	0.64-2.03	0.64	0.71	0.17-2.74	0.63
Pneumonia 2-3	1.25	0.64-2.41	0.51	0.92	0.33-2.52	0.88
Pneumonia 0-3	1.29	0.81-2.03	0.28	0.76	0.29-1.94	0.58
Cold 0-1	1.03	0.94-1.12	0.54	0.94	0.77-1.14	0.53
Cold 1-2	1.03	0.94-1.13	0.49	0.95	0.75-1.21	0.68
Cold 2-3	0.97	0.89-1.06	0.49	0.89	0.71-1.10	0.28
Cold 0-3	1.01	0.94-1.08	0.86	0.92	0.77-1.11	0.41
Tonsillitis 0-1	1.69	1.02-2.80	**0.04**	2.39	0.62-10.9	0.22
Tonsillitis 1-2	1.57	1.11-2.23	**0.01**	1.91	0.74-5.21	0.19
Tonsillitis 2-3	1.45	1.01-2.08	**0.04**	1.25	0.49-3.25	0.64
Tonsillitis 0-3	1.51	1.11-2.03	**0.008**	1.62	0.70-3.85	0.26
Acute otitis media 0-1	0.89	0.74-1.06	0.20	0.84	0.56-1.25	0.40
Acute otitis media 1-2	0.91	0.76-1.09	0.32	0.89	0.59-1.33	0.56
Acute otitis media 2-3	0.97	0.79-1.18	0.74	0.84	0.56-1.26	0.41
Acute otitis media 0-3	0.90	0.78-1.04	0.17	0.83	0.61-1.13	0.25
